# The Biofilm Inhibition Properties of Glucosamine Gold Nanoparticles in Combination with Meropenem against *Pseudomonas aeruginosa* on the Endotracheal Tube: A Model of Biofilm-Related Ventilator-Associated Pneumonia

**DOI:** 10.3390/ma17071604

**Published:** 2024-03-31

**Authors:** Dewi Santosaningsih, Yuanita Mulyastuti, Soeyati Poejiani, Rilia F. Putri, Liliana Dewi, Hisanifa Arifani, Yatim L. Ni’mah, Afaf Baktir

**Affiliations:** 1 Department of Clinical Microbiology, Faculty of Medicine, Universitas Brawijaya, Malang 65142, Indonesia; yuanmic@ub.ac.id (Y.M.); ucik.fk@ub.ac.id (S.P.); 2 Department of Clinical Microbiology, Dr. Saiful Anwar Hospital, Malang 65112, Indonesia; 3 Magister of Chemistry Study Program, Faculty of Science and Technology, Universitas Airlangga, Surabaya 60115, Indonesia; rilia.faradini.putri-2023@fst.unair.ac.id; 4 School of Medicine, Faculty of Medicine, Universitas Brawijaya, Malang 65142, Indonesia; lilyanaad10@student.ub.ac.id (L.D.); hisanifaarifani@student.ub.ac.id (H.A.); 5 Department of Chemistry, Faculty of Science and Data Analytics, Institut Teknologi Sepuluh Nopember, Surabaya 60111, Indonesia; yatimnikmah@gmail.com; 6 Department of Chemistry, Faculty of Science and Technology, Universitas Airlangga, Surabaya 60115, Indonesia

**Keywords:** biofilm, glucosamine nanoparticles, *Pseudomonas aeruginosa*

## Abstract

Biofilm-related infections play a significant role in the development and persistence of ventilator-associated pneumonia. *Pseudomonas aeruginosa* (*P. aeruginosa*) frequently causes biofilm-related infections associated with ventilator tubing. Glucosamine gold nanoparticles (AuNPs) may exhibit antibiofilm properties; however, more studies, including combinatorial therapy with antibiotics, are needed to explore their potential applications in clinical settings. This study aims to investigate the biofilm inhibition properties of glucosamine AuNPs in combination with meropenem against *P. aeruginosa* ATCC 9027 on the endotracheal tube. A biofilm inhibition assay of glucosamine AuNPs at 0.02 mg/mL, both singly and in combination with meropenem at 1 mg/mL, was carried out against *P. aeruginosa* ATCC 9027 on an endotracheal tube using the tissue culture plate method. Scanning electron microscopy was performed for visualization. Glucosamine AuNPs at 0.02 mg/mL combined with meropenem at 1 mg/mL showed greater biofilm inhibition (72%) on the endotracheal tube than glucosamine nanoparticles at 0.02 mg/mL alone (26%) (*p* = 0.001). The scanning electron microscopic visualization revealed that the untreated *P. aeruginosa* biofilm was denser than the glucosamine nanoparticles-treated biofilm, whether combined with meropenem or using glucosamine nanoparticles alone. The combination of glucosamine AuNPs and meropenem may have the synergistic effect of inhibiting biofilm production of *P. aeruginosa* on the endotracheal tubes of patients with mechanical ventilation. Conducting additional experiments to explore the impact of combining glucosamine-coated gold nanoparticles (AuNPs) with meropenem on the inhibition of biofilm production by clinical *P. aeruginosa* isolates would be beneficial.

## 1. Introduction

Biofilm infections involving *Pseudomonas aeruginosa* (*P. aeruginosa*) present a complex and persistent problem in clinical settings [1,2]. As an opportunistic pathogen, *P. aeruginosa* is known for its remarkable ability to form robust biofilms, which are organized groups of bacteria surrounded by a matrix they produce themselves [3,4,5]. This matrix provides essential structural support and protection, enabling the bacteria to adhere to surfaces, evade hosts’ immune responses, and exhibit resistance to conventional antimicrobial therapies [5,6].

The challenges posed by *P. aeruginosa* biofilm infections are multifaceted. One key challenge is the inherent antibiotic resistance displayed by bacteria within biofilms. This resistance is attributed to limited drug penetration into the biofilm matrix and the altered physiological states of bacteria residing within the biofilm [7]. Consequently, conventional antibiotic treatments often prove ineffective against biofilm-associated infections [2].

A notable aspect of *P. aeruginosa* biofilm infections is their association with medical devices, such as catheters, implants, and ventilator tubes [8,9,10,11]. These devices provide ideal surfaces for biofilm formation, leading to impaired device function and an elevated risk of systemic infections [12]. Biofilm formation on ventilator tubes poses a serious risk in healthcare settings, leading to ventilator-associated pneumonia, as well as increased patient morbidity and healthcare costs [11]. Research efforts are being made that are dedicated to unraveling the molecular mechanisms underlying *P. aeruginosa* biofilm formation and investigating potential targets for therapeutic intervention [13,14,15]. Understanding the roles of quorum sensing, extracellular polymeric substance (EPS) production, and other virulence factors is paramount in devising effective strategies to disrupt biofilm development [15].

Nanoparticles have emerged as promising tools in the battle against biofilm formation due to their unique physicochemical properties and versatile applications. These nanoscale materials exhibit a range of effects that interfere with various stages of biofilm development, offering innovative strategies to combat this persistent problem and improve the efficacy of antimicrobial approaches. The effects of nanomaterials on preventing biofilm formation include physical disruption, surface modification, release of antimicrobials, disruption of quorum sensing, electrostatic interactions, mechanical stress, photothermal effects, and enzymatic degradation [16,17].

Gold nanoparticles (AuNPs) have attracted significant scientific and technological interest in the past few decades [17,18]. In comparison with other nanoparticles, AuNPs are recognized for their exceptional stability and have been synthesized in diverse shapes and structures. Additionally, they exhibit adjustable and distinctive optical properties [17,19,20]. Currently, AuNPs have been engineered for diverse applications in the medical and pharmaceutical domains. These applications encompass utilization of their antibacterial and antibiofilm properties, among others [20].

Glucosamine is a naturally occurring amino sugar that serves as a building block for various molecules within the body, including glycosaminoglycans (GAGs) and proteoglycans. In recent years, glucosamine nanoparticles have gained attention for their potential applications in various fields, including medicine and material science [15,16,17]. In the context of preventing biofilm formation, glucosamine nanoparticles have been investigated for their ability to inhibit initial bacterial adhesion. The functional groups on the surface of glucosamine can interact with bacterial cell surfaces, disrupting their attachment and colonization on surfaces. Additionally, glucosamine nanoparticles might interfere with the quorum sensing systems that play a role in biofilm formation by inhibiting bacterial communication [6,18].

Previous studies have reported the synergistic antibiofilm effect of colistin + meropenem against *Myroides odoratimimus*, and that of a combination of liquid crystal nanoparticles + tobramycin against *P. aeruginosa* [21,22]. León-Buitimea A. et al. described a potential combination therapy using antibiotics and other antimicrobial agents, including nanoparticles, to combat antibiotic-resistant bacteria [23]. The combination of antibiotics and AuNPs, particularly those functionalized with glucosamine, has the potential to exhibit synergistic antibiofilm effects. This approach involves leveraging the advantages of both antibiotics and AuNPs to enhance antibiofilm activity and overcome limitations associated with conventional antibiotic therapies. However, the effects of the conjugation of glucosamine phosphate and AuNPs (GlcN-AuNPs)—alone and in combination with meropenem—on *P. aeruginosa* biofilms are still unclear.

We aimed to investigate the effectiveness of GlcN-AuNPs, both alone and combination with meropenem, in preventing biofilm formation of *P. aeruginosa* ATCC 9027 on the endotracheal tube, serving as a model of ventilator-associated pneumonia. Ex vivo assessment of glucosamine nanoparticles’ effectiveness against *P. aeruginosa* biofilm was conducted using the tissue culture plate method and scanning electron microscopy.

## 2. Materials and Methods

### 2.1. Endotracheal Tube Pieces Preparation

The package of endotracheal tube (Life Resources, Zhanjiang, China) was opened within the biological safety cabinet. The endotracheal tube was cut, with a sterile scalpel, to around 0.5 cm per piece. A piece of sterile endotracheal tube was placed into an individual well of tissue culture plate for further experiment [17].

### 2.2. Bacterial Isolate

*P. aeruginosa* ATCC 9027 was used in this study. The isolates from fresh agar plates were inoculated in 5 mL of trypticase soy broth (Oxoid, Basingstoke, UK) and were kept for incubation at 37 °C for 24 h. The inoculum, measured at 10^8^ CFU/mL by spectrophotometry, was used for the biofilm formation assay on the endotracheal tube.

### 2.3. AuNPs Preparation

The preparation of AuNPs with average sizes of 10 nm was conducted as previously described, with slight modifications [24,25]. Briefly, 1 mL of 0.1% *m*/*v* HAuCl_4_.3H_2_O and 49 mL water were added to 4 mL of 0.02%, 0.04%, and 0.06% (*m*/*v*) Na_3_-citrate solution, with continuous stirring at 60 °C and 800 rpm for 45 min. Subsequently, the mixture was cooled to room temperature before the addition of glucosamine phosphate.

### 2.4. Conjugation of Glucosamine Phosphate and AuNPs (GlcN-AuNPs)

The different concentrations of GlcN-AuNPs were generated as previously described [25]. Appropriate amounts of glucosamine phosphate (Sigma-Aldrich, St. Louis, MO, USA) were added into a cylindrical flask with a flat bottom containing 5 mL of AuNPs at a designated flow rate (0.150 mL/min). The mixture was stirred at 800 rpm at room temperature. The final concentrations of GlcN-AuNPs were 0.008%, 0.012%, 0.016%, 0.020%, and 0.024%. Ultraviolet–visible spectrophotometry (ThermoScientific Genesys 150, Thermo Fisher Scientific, Waltham, MA, USA) and transmission electron microscopy (TEM) (JOEL JEM-1400, Peabody, MA, USA) of GlcN-AuNPs were performed prior to usage for characterization and quality assurance purposes. The TEM images were obtained at the TEM service unit in the Department of Chemistry, Faculty of Mathematics and Natural Sciences, Gadjah Mada University, Yogyakarta, Indonesia.

### 2.5. Ex Vivo Assessment of GlcN-AuNPs against P. aeruginosa ATCC 9027 Biofilms

The tissue culture plate method was carried out to investigate the biofilm formation of *P. aeruginosa* ATCC 9027 on the endotracheal tube pieces [26]. The effect of GlcN-AuNPs, either alone or in combination with meropenem, on early biofilms was evaluated. Therefore, the plates underwent a two-step incubation process [4].

Pieces of ETT were placed in individual wells of 24-well flat bottom polystyrene (Biologix Europe GmbH, Hallbergmoos, Germany). Individual wells of 24-well flat bottom polystyrene were filled with 200 μL of 10^8^ CFU/mL *P. aeruginosa* ATCC 9027 inoculums and 200 μL trypticase soy broth–glucose medium. The uninoculated medium, serving as the negative control, was added to each well to ensure that the medium was sterile. After the plates were incubated for eight hours at 37 °C (first incubation), the broth medium was carefully pipetted out of the wells. Plates were washed twice with 200 μL of phosphate buffer saline (pH 7.2). The ETT was moved to new individual wells of 24-well flat bottom polystyrene. The individual wells were filled with 200 μL of trypticase soy broth (without glucose) for further experiments described in Table 1 [4].

Plates then were incubated at 37 °C for 24 h (second incubation). After 24 h of incubation, plates were washed twice with 200 μL of phosphate buffer saline (pH 7.2) and incubated at 37 °C for an hour. The plates were stained with 200 μL of 0.1% crystal violet for 10 min. Excess stain was removed by washing twice with deionized water and the plates were kept for drying. Next, 200 μL of 33% glacial acetic acid was added to the wells. The optical densities (OD) of the isolates were determined using a micro-ELISA auto reader (BIORAD, Hercules, CA, USA) at a wavelength of 570 nm. The experiment was performed in quadruples per group. The analysis of optical density data for identifying biofilm formation is displayed in Table 2. Additionally, the percentage of biofilm inhibition was determined using the following formula [22]:

% inhibition = 100 − (OD570 sample/OD570 untreated group × 100).

### 2.6. Quantification of Bacterial Biofilms

The quantification of bacterial biofilms was conducted as previously described [28]. Briefly, the biofilm was extracted from endotracheal tube (ETT) pieces using vortex and sonication. The sonicate was concentrated by centrifugation at 3100× *g* for 10 min and the resulting pellet was resuspended in 1 mL PBS. Serial dilutions, including neat sonicate, were plated on LB agar using the 20 μL drop method in triplicate and then incubated at 37 °C for 14–18 h. After incubation, the number of colonies per 20 μL drop was counted. The quantification of biofilm bacteria utilized the formula of CFU/cm = (average number of colonies for a dilution) × 50 × dilution factor.

### 2.7. Scanning Electron Microscope

Scanning electron microscope preparation was conducted following the previous described method [29]. Initially, the suspensions containing a piece of ETT in each well of the tissue culture microplate were discarded. Subsequently, each ETT piece underwent two gentle rinses with 1% sterile phosphate-buffered saline (Merck, Rahway, NJ, USA) and was fixed with 2.5% glutaraldehyde for 2 h. The fixed ETT pieces were then washed again with PBS and dehydrated through a series of graded ethanol solutions. Finally, the ETT pieces were removed from the wells, dried overnight, and coated in gold before imaging. The examination was performed using a scanning electron microscope (Hitachi TM 3000, Tokyo, Japan) at the Laboratory of Bioscience, Brawijaya University, Malang, Indonesia.

## 3. Results

### 3.1. Characterization of GlcN-AuNPs

**Figure 1:**
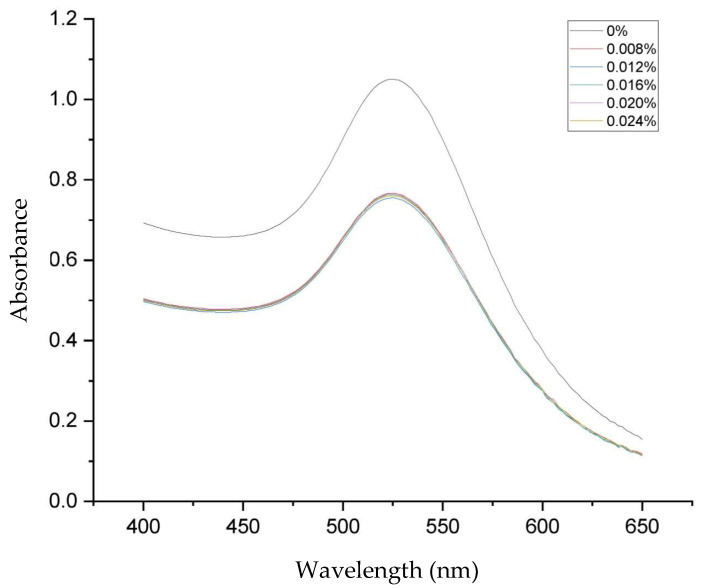
Visible absorption spectra for AuNPs without glucosamine (0%) and functionalization of GlcN-AuNPs in different concentrations (0.008%, 0.012%, 0.016%. 0.020%, and 0.024% *m*/*v*).

**Figure 2:**
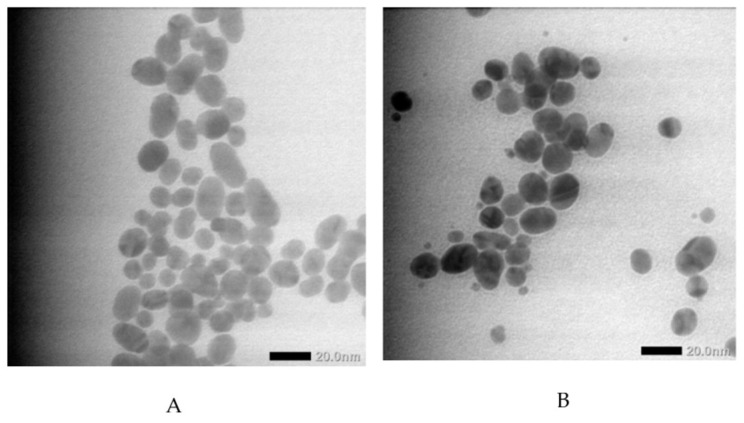
Transmission electron micrographs of (**A**) AuNPs and (**B**) GlcN-AuNPs showing size distributions and shapes.

Figure 1 displays the ultraviolet–visible spectra of both AuNPs and GlcN-AuNPs, revealing a distinctive absorption band in the range of approximately 500–550 nm. The AuNPs (0%) showed an absorption band at 525 nm; this presence signifies the formation of AuNPs. The functionalization of GlcN-AuNPs in different concentrations, including 0.008%, 0.012%, 0.016%, 0.020%, and 0.024% (*m*/*v*), resulted in similar absorption bands at 525 nm, reflecting the similar sizes, shapes, and surfaces of the nanoparticles. In addition, the morphologies and particle sizes of AuNPs and GlcN-AuNPs were characterized by TEM. Both AuNPs and GlcN-AuNPs were found to have a spherical shape and exhibited an average size of less than 100 nm (Figure 2).

### 3.2. Biofilm Formation of P. aeruginosa ATCC 9027 on an Endotracheal Tube

**Figure 3:**
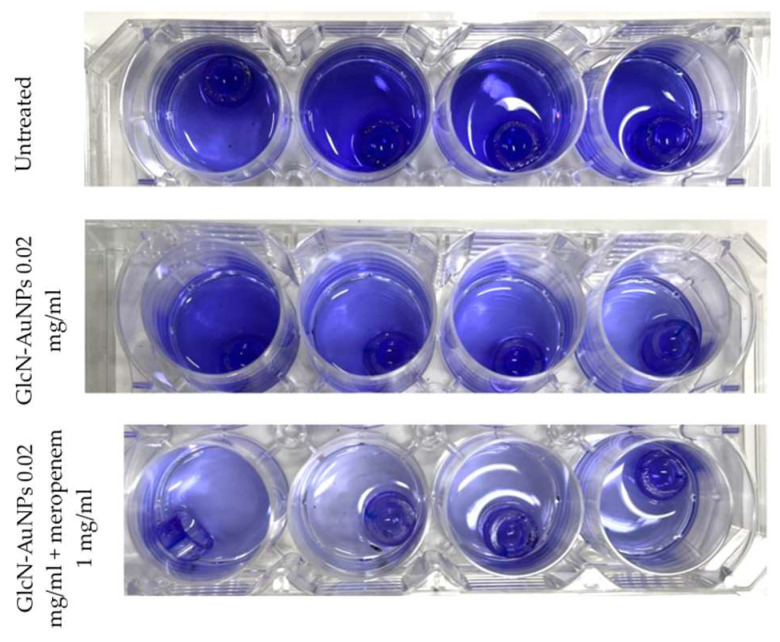
Detection of biofilm formation by *P. aeruginosa* ATCC 9027 on endotracheal tube pieces using tissue culture plate method (four replications per group) before acid was added.

The detection of biofilm formation by *P. aeruginosa* ATCC 9027 on endotracheal tube pieces using the tissue culture plate method shows different intensities among treatment groups (Figure 3). In concordance with the interpretation of the optical density results presented in Table 3, the untreated group (first row) showing high intensity indicates strong biofilm production. After exposure to GlcN-AuNPs alone and in combination with meropenem (second and third row), the intensity of biofilm formation by *P. aeruginosa* ATCC 9027 was lower, exhibiting moderate biofilm production.

**Figure 4:**
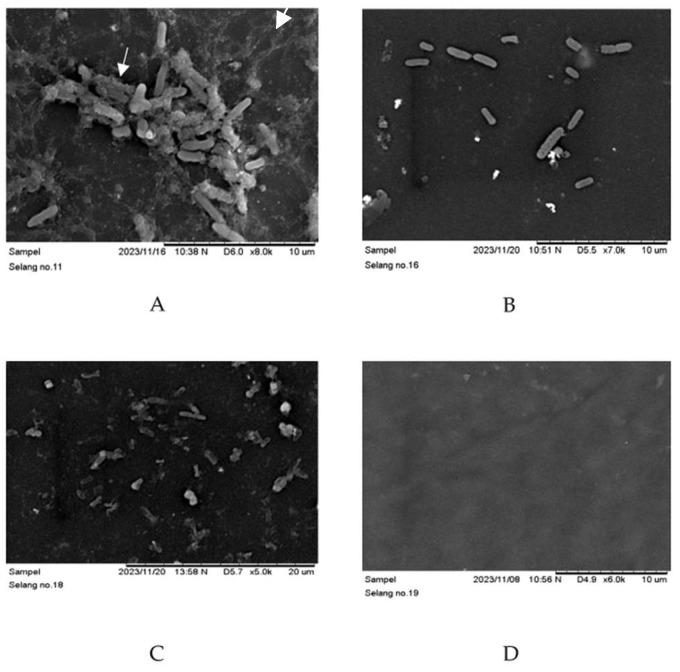
Biofilm formation of *P. aeruginosa* ATCC 9027 on endotracheal tube pieces, observed by scanning electron microscopy. (**A**) = untreated *P. aeruginosa* ATCC 9027; white arrow shows bacterial cells covered by extra polysaccharides matrix (**B**) = *P. aeruginosa* ATCC 9027 treated with GlcN-AuNPs 0.02 mg/mL alone; (**C**) = *P. aeruginosa* ATCC 9027 treated with GlcN-AuNPs 0.02 mg/mL + meropenem 1 mg/mL; (**D**) = negative control; white arrow shows bacterial cells covered by extrapolysaccharides matrix.

In addition, endotracheal tube pieces exposed to untreated *P. aeruginosa* ATCC 9027 demonstrated biofilm production, with the biofilm manifesting as confluent sheets of cells within a dense extracellular matrix observed by scanning electron microscopy (Figure 4A). In contrast, the negative control tube containing trypticase soy broth media showed no evidence of biofilm formation upon scanning electron microscopy examination (Figure 4D). Figure 4B displays the distribution of intact planktonic cells not covered by an extracellular matrix, indicating the inhibition of biofilm formation of *P. aeruginosa* ATCC 9027 after exposure to GlcN-AuNPs 0.02 mg/mL alone. The combination of GlcN-AuNPs 0.02 mg/mL and meropenem 1 mg/mL applied to the ETT pieces resulted in a simultaneous effect. The SEM image shows damage to the planktonic cells, and the extracellular matrix sheet is absent (Figure 4C).

### 3.3. Biofilm Inhibition Activities of GlcN-AuNP, Both Alone and in Combination with Meropenem, against P. aeruginosa ATCC 9027 on the Endotracheal Tube

**Figure 5:**
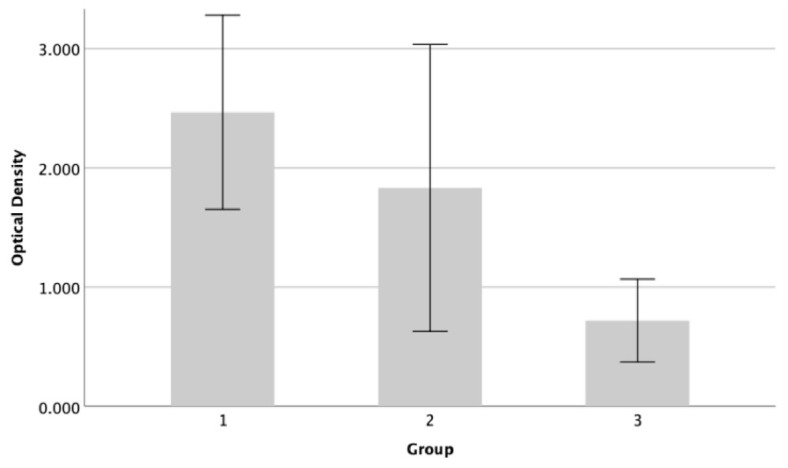
The biofilm-forming activity of *P. aeruginosa* ATCC 9027. The assessment of biofilm formation was conducted using the microtiter plate technique and staining with crystal violet. The graph displays the average ± standard deviation of biofilm formation derived from three groups. Group 1 = untreated; group 2 = GlcN-AuNP 0.02 mg/mL; group 3 = GlcN-AuNPs 0.02 mg/mL + meropenem 1 mg/mL.

The results demonstrated a decrease in biofilm production of *P. aeruginosa* 9027 on the endotrachael tube after exposure to GlcN-AuNPs, either alone or in combination with meropenem (Figure 5). The percentage of biofilm inhibition was 26% and 71% after exposure to GlcN-AuNPs alone and in combination with meropenem, respectively. *P. aeruginosa* ATCC 9027 exhibited the lowest level of biofilm formation, significantly decreased after exposure to the combination of GlcN-AuNPs and meropenem (F value = 16.832; *p* = 0.001). The Tukey post-hoc test revealed a significant decrease in the biofilm formation level of *P. aeruginosa* 9027 when treated with the combination of GlcN-AuNPs and meropenem, compared to both the untreated group and those treated with GlcN-AuNPs alone (Table 3).

Quantification of *P. aeruginosa* ATCC 9027 biofilms on the cultivation media showed decreases in the colony-forming unit counts of bacterial biofilms after exposure to either GlcN-AuNPs 0.02 mg/mL alone (99%) or GlcN-AuNPs 0.02 mg/mL combined with meropenem 1 mg/mL (95%). However, the CFU count was not significantly different after exposure to GlcN-AuNPs 0.02 mg/mL alone compared to GlcN-AuNPs 0.02 mg/mL combined with meropenem 1 mg/mL (*p* = 0.042) (Table 4).

## 4. Discussion

Ventilator-associated pneumonia is defined as pneumonia that develops in patients undergoing mechanical ventilation via an ETT for over 48 h in the hospital. The formation of a biofilm on the ETT significantly contributes to the occurrence of VAP [11]. This type of pneumonia affects 25–56% of all mechanically ventilated patients [30].

Ventilator-associated pneumonia is mainly caused by multidrug-resistant and extremely drug-resistant strains of *P. aeruginosa*, leading to high rates of treatment failure. The effectiveness of antibiotics such as aminoglycosides, quinolones, and β-lactams are limited by the rapid formation of *P. aeruginosa* biofilms on endotracheal tubes, which are a crucial virulence factor and make bacterial elimination more challenging [31,32].

Meropenem, a β-lactam antibiotic, is highly effective against multidrug-resistant *P. aeruginosa* [29]. Nevertheless, there is a global trend of increased carbapenem resistance among these organisms [33].

Nanomaterials with strong antimicrobial properties are potential alternatives to conventional antibiotics, as they bypass common antibiotic resistance mechanisms [34]. They inhibit biofilm formation through various mechanisms such as physical disruption, surface modification, antimicrobial release, interference with quorum sensing, electrostatic interactions, mechanical stress, photothermal effects, and enzymatic degradation [16,17]. In addition, there have been reports on the synergistic effect of combining nanomaterials with antibiotics to prevent biofilm formation [23]. A prior study documented the benefit of the antimicrobial and antibiofilm effects of meropenem and ZnO nanoparticles in protecting a cornea rat model from pseudomonas-induced keratitis [29].

To our knowledge, this study is the first to experiment with synthesizing glucosamine phosphate with gold nanoparticles (GlcN-AuNPs) and applying the resulting material simultaneously with meropenem to inhibit biofilm formation on the endotracheal tube. Gold nanoparticles are recognized for their significant potential in biomedical applications, including drug delivery, imaging, and diagnostics, due to their ease of preparation, surface reactivity, and unique optical properties [35]. Gold is a generally non-toxic nanomaterial; however, the substances employed in its preparation and modification may have toxic properties. However, at specific concentrations, gold nanoparticles exhibit antibacterial effects without toxicity to normal cells [19]. In addition, the compact size of AuNPs and their biocompatibility with biological systems, minimal toxicity, and ability to combine different molecular functionalities simultaneously make them attractive for use in biomedical applications involving therapy and sensing [35].

In this study, we found that AuNPs conjugated with glucosamine phosphate effectively inhibited biofilm formation by *P. aeruginosa* ATCC 9027 on the endotracheal tube. This finding is consistent with a previous study that highlighted the effectiveness of AuNPs in combating biofilm-related infections caused by *P. aeruginosa* PAO1 [36]. Experimental evidence suggests that the glucose subunit in glucosamine phosphate plays a crucial role, stimulating the generation of intracellular NAG-6-P, which inactivates the regulator NagC and reduces biofilm formation [37,38].

Moreover, this study demonstrated that combining GlcN-NPs and meropenem effectively reduced biofilm formation by *P. aeruginosa* ATCC 9027 on the endotracheal tube. The combination was nearly three times more effective than GlcN-NPs alone (Figure 5). The statistical analysis, using Tukey’s post-hoc test, revealed no significant difference in mean optical density between the untreated group (*p* = 2.466 ± 0.406) and GlcN-AuNP 0.02 mg/mL (*p* = 1.832 ± 0.601). However, a significant difference was observed between the untreated group and GlcN-AuNPs 0.02 mg/mL + meropenem 1 mg/mL (*p* = 0.720 ± 0.173). The combination of nanomaterials and antibiotics could potentially contribute to enhancing the activity of biofilm inhibition against *P. aeruginosa* ATCC 9027 [29]. Nevertheless, further research is needed to understand the impact of GlcN-AuNPs and meropenem on clinical *P. aeruginosa* isolates from mechanically ventilated patients.

The quantification of bacterial biofilms showed that either GlcN-NPs alone or GlcN-AuNPs 0.02 mg/mL combined with meropenem 1 mg/mL could reduce the bacterial load of *P. aeruginosa* ATCC 9027 in the biofilm compared to the untreated group (*p* = 0.042). However, the colony-forming unit count was not significantly different between the treated groups. It is suggested that the combination of nanomaterials and antibiotics did not reduce the bacterial load in the biofilm more than nanomaterials alone. According to the previous study, the analysis of bacterial biofilm using the sonication method is influenced by the detachment of the bacterial cells from the surface, the count of colony-forming units, and the stage of detached bacterial biofilms. Therefore, quantification is limited to viable and cultivable bacterial cells [28].

This study had certain limitations. First, the antibiofilm activity of meropenem 1 mg/mL alone was not evaluated in this study, therefore the synergistic effect with meropenem could not be confirmed. Second, the structure of the biofilm involved was not described quantitively, including analysis of thickness variability, roughness coefficient, substratum coverage, and surface-to-volume ratio [39]. Further experiments using confocal imaging and the COMSTAT program are necessary to validate the results.

## 5. Conclusions

In summary, our study highlights the potential of employing combination therapies involving GlcN-AuNPs 0.02 mg/mL in conjunction with meropenem 1 mg/mL, as opposed to relying solely on antibiotics. Our findings underscore the enhanced antibacterial and antibiofilm efficacy achieved with the combination of GlcN-AuNPs and meropenem, presenting a successful strategy to counter the emerging resistance of *P. aeruginosa* to carbapenems. This approach aims to mitigate the risk of increased resistance, thereby reducing the higher morbidity and mortality resulting from treatment failures. Further investigation with quantitative analysis is necessary to confirm the synergistic effect between GlcN-AuNPs and meropenem.

## Figures and Tables

**Table 1:** The ex vivo assessment of GlcN-AuNPs against *P. aeruginosa* biofilm.

Group	Treatment
1	*P. aeruginosa* ATCC 9027 (untreated)
2	*P. aeruginosa* ATCC 9027 + GlcN-AuNPs 0.02 mg/mL
3	*P. aeruginosa* ATCC 9027 + GlcN-AuNPs 0.02 mg/mL + meropenem 1 mg/mL

**Table 2:** Interpretation of optical density data for detection of biofilm formation [27].

Average OD Value	Interpretation
OD ≤ ODc	No biofilm formation
ODc < OD ≤ 2 × ODc	Weak biofilm formation
2 × ODc < OD ≤ 4 × Odc	Moderate biofilm formation
4 × ODc < OD	Strong biofilm formation

Notes: all OD values were measured at 570 nm; ODc = average OD of negative control + 3 × standard deviation of the negative controls; Odc = optical density cutoff value.

**Table 3:** Biofilm activity assay of *P. aeruginosa* ATCC 9027 treated with GlcN-AuNPs.

Group	Replication	OD	Mean ± SD	Interpretation
Untreated	1	1.863	2.466 ± 0.406 ^a^	Strong biofilm formation
	2	2.724		
	3	2.584		
	4	2.695		
GlcN-AuNPs 0.02 mg/mL	1	2.618	1.832 ± 0.601 ^a^	Moderate biofilm formation
	2	1.887		
	3	1.648		
	4	1.176		
GlcN-AuNPs 0.02 mg/mL + meropenem 1 mg/mL	1	0.908	0.720 ± 0.173 ^b^	Moderate biofilm formation
	2	0.533		
	3	0.620		
	4	0.819		

One-way ANOVA analysis: *p*-Value = 0.001, ^a^ and ^b^ indicate results that are significantly different according to Tukey’s post-hoc test. OD = optical density; SD = standard deviation.

**Table 4:** Quantification of bacterial biofilms from *P. aeruginosa* ATCC 9027 treated with GlcN-AuNPs on the surface of endotracheal tube pieces.

Group	Replication	CFU/cm	Mean ± SD
Untreated ^a^	1	50.1 × 10^6^	46.4 × 10^6^ ± 5.5 × 10^6^
	2	49.1 × 10^6^	
	3	40.1 × 10^6^	
GlcN-AuNPs 0.02 mg/mL ^b^	1	11.2 × 10^4^	44.0 × 10^3^ ± 59.7 × 10^3^
	2	2.0 × 10^4^	
	3	0	
GlcN-AuNPs 0.02 mg/mL + meropenem 1 mg/mL ^b^	1	54.1 × 10^3^	20.2 × 10^5^ ± 19.7 × 10^5^
	2	20.1 × 10^5^	
	3	40.0 × 10^5^	

Kruskal–Wallis analysis: *p*-Value = 0.042, ^a^ and ^b^ indicate results that are significantly different. CFU = colony-forming unit; SD = standard deviation.

## Data Availability

Data are contained within the article.
